# Wheat Peptides as Catalysts for Athletic Performance Improvement in Cross-Country Skiers: A Randomized Controlled Trial

**DOI:** 10.3390/metabo14100538

**Published:** 2024-10-07

**Authors:** Mai Xiang, Qi Han, Yue Chen, Shenglin Duan, Xiaofeng Han, Xuemei Sui, Chaoxue Ren, Qirong Wang

**Affiliations:** 1 Sports Nutrition Center, National Institute of Sports Medicine, Beijing 100029, China; michael9789@126.com (M.X.); hanqi0418@163.com (Q.H.); cy13820037320@126.com (Y.C.); 2 Key Laboratory of Sports Nutrition, General Administration of Sport, Beijing 100029, China; 3 College of Exercise Science, Beijing Sport University, Beijing 100084, China; 4 Beijing Key Laboratory of the Innovative Development of Functional Staple and the Nutritional Intervention for Chronic Disease, Beijing 100015, China; dslbeijing@163.com; 5 China National Research Institute of Food & Fermentation Industries, Beijing 100015, China; hanxf2001@126.com; 6 Department of Exercise Science, Arnold School of Public Health, University of South Carolina, Columbia, SC 29208, USA; msui@mailbox.sc.edu; 7 School of Exercise & Health Science, Xi’an Physical Education University, Xi’an 710068, China

**Keywords:** athletic performance, cross-country skier, energy bar, wheat peptide, protein, metabolomics

## Abstract

Objectives: This study investigated the efficacy of wheat peptide supplementation compared to regular proteins in elite cross-country skiers, providing insights into the metabolic and performance effects of these supplements in order to guide athletes in selecting optimal energy sources for training and competition. Methods: Nineteen healthy male cross-country skiers were enrolled and assigned to either the peptide group (PEP, *n* = 9) or the protein group (PRO, *n* = 10). A four-week intervention study involving supplementation with wheat peptides/regular proteins was conducted, and pre- and post-intervention assessments were performed to evaluate exercise capacity and metabolic profiles. Results: The study found that the PEP group and the PRO group showed distinct within-group effects on exercise performance. The PEP group demonstrated improved aerobic capacity, including better performance in 10 km roller skating, an increased lactate threshold, and reduced resting blood lactate levels. The PRO group enhanced anaerobic capacity, such as improved sprint time, hexagon test performance, and lactate clearance. Metabolomic analysis revealed specific metabolic pathways affected in each group, with the PEP group showing impacts on the α-linolenic acid pathway and the PRO group on ketone body synthesis and degradation as well as vitamin B6 metabolism. Conclusions: Our findings indicate that wheat oligopeptides and regular proteins have comparable effects on exercise performance. However, the wheat peptides may offer greater advantages in enhancing aerobic capacity. No significant variations were observed in blood metabolite profiles between the two groups, but distinct metabolic pathways exhibited different responses.

## 1. Introduction

Cross-country skiing is a physically demanding endurance sport that requires both upper and lower body strength and endurance [1,2]. Elite cross-country skiers have exceptional aerobic and anaerobic capabilities, explosive power, and efficient skiing technique [2,3,4,5]. Nutrition plays a crucial role in supporting their performance, with protein being an important energy source and key for muscle remodeling [6,7].

Proteins serve as an important energy substrate during endurance exercise, contributing around 10% of energy demands [8], while also serving as a pivotal substrate for skeletal muscle remodeling [9]. Athletes frequently utilize protein supplements to augment muscle mass and strength for enhanced performance. Studies have shown that consuming protein after endurance exercise can optimize muscle protein synthesis and aid recovery [8,10,11]. Peptides, which are smaller protein fragments, have unique advantages in digestion and absorption compared to whole proteins [12,13]. They are rapidly absorbed by the intestines and provide amino acids more efficiently [14,15]. Some peptides have been found to stimulate muscle protein synthesis [16], increase muscle strength [17], and improve recovery [18].

The effects of peptides on endurance performance are still being explored, previous research suggests that they may have positive impacts [19,20,21,22]. For instance, research by Lollo et al. has demonstrated potential benefits of protein hydrolysates (hydrolyzed whey protein) over intact whey protein in reducing markers of muscle damage in soccer players [22]. Furthermore, plant-derived peptides have shown benefits for endurance [23,24], but studies specifically examining the effects of protein hydrolysates or oligopeptides in elite athletes are limited. However, a recent review suggests there is currently insufficient evidence to support protein hydrolysates having a superior advantage over intact non-hydrolyzed proteins in muscle synthesis and recovery [25].

This study aims to compare the metabolic profiles and exercise performance of athletes consuming wheat oligopeptides versus regular proteins. By investigating the efficacy of wheat peptide supplementation, the research will contribute to our understanding of how wheat peptides can influence endurance exercise. The results will provide valuable insights for athletes in selecting suitable energy replenishment strategies during training and competition.

## 2. Materials and Methods

### 2.1. Participants

Nineteen healthy male cross-country skiers were recruited, meeting the criteria of being free from cardiorespiratory, metabolic, and musculoskeletal diseases, and having no smoking, alcohol consumption, recent infections, or injuries. Baseline measurements such as height, weight, and body composition (InBody 570, In Body, Cerritos, CA, USA) were taken (see Table 1). To ensure consistency and account for daily fluctuations in body fluid distribution, measurements were conducted in the morning before breakfast, with participants wearing only their underwear for standardization purposes. This study has been retrospectively registered in the Chinese Clinical Trials Registry (registration number ChiCTR2400086034, approved on 24 June 2024).

Participants were randomly assigned to either the intervention group (PEP, *n* = 9) or the control group (PRO, *n* = 10) using a blocked randomization process with a block size of 5. This approach ensured balanced group representation as participants were recruited. Randomized combinations of treatment assignments were pre-generated and sequentially assigned to participants, minimizing allocation bias and enhancing the reliability of the group comparisons.

### 2.2. Study Design

**Figure 1:**
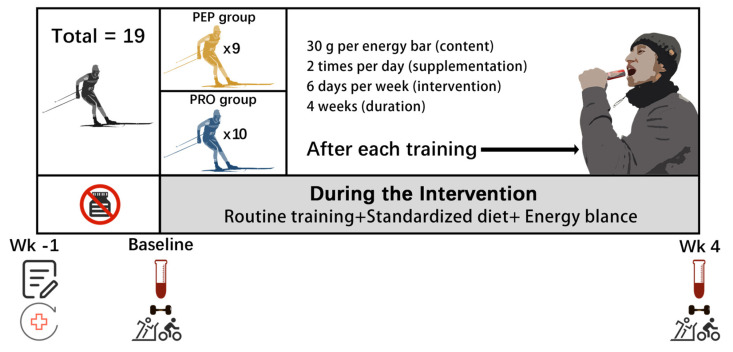
General experimental design.

The brief description of the experimental procedure is presented in Figure 1. The study employed a four-week double-blind paired design. Participants in both groups consumed energy bars containing wheat peptides or regular proteins (control) after morning and afternoon training sessions. Blood samples were collected and tests were performed before and after the intervention period.

During the intervention period, athletes underwent concentrated training at the Shaanxi Ankang National Snow Sports Training Base in China, which is situated at an altitude of 1000 m.

The athletes are currently undergoing the preparatory phase of a periodized training regimen, which involves a gradual increase in training intensity. They were asked to maintain their usual training routine to accurately reflect their typical exercise loads. This holistic training strategy encompasses a well-rounded mix of aerobic endurance, interval sessions, and high-intensity interval training, aligning with their standard training practices. Additional information on the training program is detailed in the Supplementary Materials Table S4.

The participants followed a standardized diet consisting of three meals daily, with their caloric intake primarily from carbohydrates (60–65%), fats (25–30%), and proteins (12–15%). The protein sources were diverse, including lean meats such as chicken, beef, and fish as well as dairy and eggs for animal-based proteins, complemented by plant-based options such as legumes and grains. Dietary monitoring was conducted thrice weekly, with the weekly assessments now accessible in the Supplementary Materials Table S1.

We conducted daily monitoring of the athletes, which included assessments of their training duration and average heart rate during workouts. Additionally, we implemented nighttime sleep monitoring, recording metrics such as pre-sleep blood pressure, total sleep duration, deep sleep duration, and average heart rate during sleep (all data can be found in the Supplementary Materials Table S2 and S3).

On the day preceding the tests, participants were advised to refrain from exercising and observe a fast after dinner, and they were permitted to drink water only.

### 2.3. Supplementation Protocol

Prior to the experiment, participants’ average daily dietary protein intake was 112.77 g, which fell short of the recommended intake of 2 g/kg body weight. The average weight of the subjects was 62.6 kg, suggesting a standard protein intake of 125.2 g. Thus, an additional 12.43 g of specialized protein was required daily.

Wheat peptides and regular proteins were both supplemented in the form of energy bars, which were supplied by the Beijing Key Laboratory of Creation of Functional Staple Foods and Nutritional Intervention for Chronic Diseases. Regardless of the presence of wheat peptides, the energy bars have consistent values of calories (1890 kcal), carbohydrates (34.2 g/100 g), fat (23.5 g/100 g), and protein content (19.7 g/100 g). In this study, we replaced the regular protein component with wheat oligopeptides at a concentration of 6.1 g/100 g and incorporated them into the energy bars. Based on previous calculations, to meet the protein requirements, each participant needed to consume two energy bars (30 g each) per day to supplement additional peptides or proteins. The energy bars were distributed on-site and consumed immediately 30 min after training, with standardized packaging, by the testing personnel.

### 2.4. Blood Collection

The phlebotomists arrived at the athletes’ dormitories at 6:30 am and collected fasting blood samples via venepuncture. Dry anticoagulant was added slowly along the walls of the tubes containing the blood samples, and the tubes were gently agitated to ensure complete and uniform mixing. Then, the samples were immediately placed into a freezer for storage. Once sampling was complete for all athletes, the blood samples were transported to the laboratory for analysis.

### 2.5. Athletic Performance Tests

#### 2.5.1. Sport-Specific Ability Tests

The following sport-specific ability tests were administered:

(1) The 30 m Sprint: The completion time for a maximum-effort 30 m sprint was recorded using TC-Timers (Brower, Sandy, USA);
(2) The Hexagonal Agility Test: The time taken to complete three full hexagonal circuits was recorded using a Seiko SVAS009 stopwatch (Seiko Instruments Inc, Tokyo, Japan);
(3) Relative Bench Press Strength: The maximum bench press weight relative to body weight was recorded;
(4) The 1500 m Run: The completion time for a 1500 m run on a standard track was recorded with a Seiko SVAS009 stopwatch (Seiko Instruments Inc, Tokyo, Japan);
(5) The 10 km Roller Skating Event: The completion time for a 10 km roller skating event was recorded with a Seiko SVAS009 stopwatch (Seiko Instruments Inc, Tokyo, Japan).

#### 2.5.2. Aerobic Capacity Tests

The 10 km roller skating results and 1500 m run time from the sport-specific ability tests were also used as indicators of aerobic capacity. Direct aerobic capacity testing was performed using treadmills (h/p/cosmos plus, Germany). The steps were as follows:

(1) Pre-exercise measurements: We measured the resting heart rate using monitors (Firstbeat, Jyväskylä, Finland), blood glucose using glucometers (Ecoing R2, Aikang Biotechnology Co., Ltd., Håangzhou, China), and blood lactate using portable lactate analyzers (EKF Lactate Scout 4, EKF Diagnostics, Cardiff, UK);
(2) Warm-up: Subjects performed a 5–10 min warm-up prior to treadmill testing;
(3) Preparation: We collected subjects’ personal details, attached heart rate monitors, and connected a respiratory mask and gas analyzer (METAMAX3B, Cortex Biophysical GmbH, Leipzig, Germany);
(4) Testing protocol: The test started with a 2 min warm-up at 6 km/h and 0% grade, followed by incremental increases in speed and grade, during which we monitored heart rate, respiratory exchange ratio (RER), and Rating of Perceived Exertion (RPE);
(5) Test termination: We ended testing when specific criteria were met, including an oxygen uptake plateau, small differences between stages, attainment of the maximum heart rate, high RER and RPE, or a subject’s inability to continue;
(6) Post-exercise measurements: We recorded post-exercise blood glucose, RPE, heart rate, and blood lactate at 0, 5, and 10 min;
(7) Data analysis: We analyzed VO_2_max, ventilatory threshold, and time to anaerobic threshold (AT), as well as heart rate, blood glucose, and lactate at AT.

#### 2.5.3. Anaerobic Capacity and Strength Tests

The 30 m sprint, hexagonal agility test, relative bench press strength, and vertical jump test from the sport-specific ability tests were also used as indicators of anaerobic capacity and strength. The vertical jump test included a squat jump (SJ) and a countermovement jump (CMJ) and was conducted using a force platform (kistler 9287C, Winterthur, Switzerland). Each subject performed three trials of SJ and CMJ, and the maximum height achieved was recorded.

In addition, the Wingate test, as a direct anaerobic capacity test, was employed to assess average power, peak power, and fatigue index. Pre- and post-test measurements of blood glucose and lactate levels were taken to analyze changes. Resting blood glucose and lactate levels were measured prior to the testing sessions.

During the Wingate test, the ergometer (POWERMAX-VII, Combi, Tokyo, Japan) load was 0.083 × body weight (kg). Power = load resistance (N) × 11.765 × revolutions. The test procedure was as follows:

(1) Subjects performed a two-minute warm-up increasing their heart rate to 150–160 bpm, including 2–3 maximum 4–8 s sprints;
(2) After two minutes of rest, subjects performed an all-out 30 s sprint against a constant load;
(3) Revolutions were recorded every 5 s during the test as times were called, and power was calculated using the formula;
(4) Post-test, the load was reduced, and subjects performed light activity for 2–3 min.

The test indices were as follows:

(1) Peak power: Highest five-second power recorded;
(2) Average power: Mean of the 6 five-second power values;
(3) Fatigue index: (Peak power−minimum power)/peak power × 100;
(4) The blood glucose level immediately after exercise and the blood lactate levels at 0, 5, 7, and 9 min after exercise.

### 2.6. Metabolomics Experiment

The samples were re-numbered at the core facility to better manage and preserve them, with this numbering used throughout the experimental analysis. The main instruments and reagents used were a refrigerated high-speed centrifuge (Centrifuge 5430, Eppendorf, Hamburg, Germany), a vortex mixer (QL-901, Qilinbeier Instrument Manufacturing Co., Beijing, China), an ultra-pure water system (Milli-Q Integral, Millipore Corporation, Burlington, MA, USA), a freeze-drying vacuum concentrator (Maxi Vacbeta, GENE COMPANY, Hong Kong, China), a tissue homogenizer (JXFSTPRP, Shanghai, China), methanol and acetonitrile (LCMS grade, Thermo Fisher Scientific, Waltham, MA, USA), ammonium acetate (17843-250G, Honeywell Fluka, Morris Plains, NJ, USA), and formic acid (50144-50 mL, DIMKA, Minneapolis, MN, USA), with water from the ultra-pure water system.

#### 2.6.1. Metabolite Extraction

Metabolites were extracted as follows:

(1) A 20 μL volume of each sample and standard was combined with 120 μL of protein precipitation solution, shaken at 1200 rpm for 30 min, and centrifuged at 18,000 g at 4 °C for 30 min;
(2) A 30 μL volume of supernatant was transferred to a 96-well plate with 20 μL of derivatization reagent and 20 μL of EDC working solution;
(3) The plate was sealed and incubated at 40 °C with shaking at 1200 rpm for 60 min;
(4) The plate was centrifuged at 4000 g at 4 °C for 5 min, after which 30 μL was transferred to a new plate with 90 μL of dilution solvent and shaken at 600 rpm for 10 min;
(5) The new plate was centrifuged at 4000 g at 4 °C for 30 min, sealed, and prepared for analysis.

#### 2.6.2. UPLC-MS Analysis

Metabolite separation and quantitative detection were performed using a Waters ACQUITY UPLC I-Class Plus (Waters Co., Milford, MA, USA) coupled to a high-sensitivity QTRAP6500 Plus mass spectrometer (SCIEX, Framingham, MA, USA).

Chromatographic conditions: The column used was a BEH C18 (2.1 mm × 10 cm, 1.7 μm, Waters). The mobile phases were 0.1% formic acid in water (Solvent A) and 30% acetonitrile (Solvent B). The following gradient was used for elution: 0–1.00 min, 5% B; 1.00–5.00 min, 5%–30% B; 5.00–9.00 min, 30%–50% B; 9.00–11.00 min, 50%–78% B; 11.00–13.50 min, 78%–95% B; 13.50–14.00 min, 95%–100% B, with a flow rate of 0.40 mL/min for the above; 14.00–16.00 min, 100% B, with a flow rate of 0.60 mL/min; 16.00–18.00 min, 5% B, with a flow rate of 0.40 mL/min. The column temperature was 40 °C.

Mass spectrometry conditions: Parameters for the ESI Turbo spray ionization source of the QTRAP 6500 Plus were as follows: ion source temperature 400 °C; ion spray voltages +4500 V/−4500 V; ion source gases GS1 and GS2 and curtain gas at 60, 60, and 35 psi, respectively. MRM methods were set up in MRM mode containing MRM transitions, collision energies, and declustering potentials for target metabolites.

Metabolite peaks were extracted and metabolites identified using the quantitative software 22.2 Skyline, with further processing of the resultant data matrix.

### 2.7. Statistical Analysis

Athletic Performance Data: Data were processed using SPSS 26.0. Descriptive statistics (mean ± SD) were used. Normality was checked using the Shapiro–Wilk test. If the data were non-normal, appropriate transformations were applied. The independent samples *t*-test was used to compare groups, while the paired samples *t*-test was used to evaluate within-group differences. Two-way repeated-measures ANOVA was used to assess dependent variable changes over time. Data violating the sphericity assumption were adjusted with the Greenhouse–Geisser correction (*p* < 0.05). Bonferroni post hoc tests were used to analyze within-group effects, and *t*-tests were used to compare specific time points between groups. Pairwise comparisons with the Bonferroni correction were conducted if no significant interaction but significant main effects were found. Partial eta squared (pη^2^) was calculated as a measure of effect size for the ANOVA, where values of 0.01, 0.06, and 0.15 correspond to small, medium, and large effects, respectively [26]. The significance level was set at *p* < 0.05.

Metabolomics Data: Data were processed using MetaboAnalyst 5.0. Data were normalized using median normalization (within groups) and normalization based on pooled/average samples (between groups). Auto-scaling pre-processing was applied. Discrimination models (PCA, OPLS-DA) were established, and permutation tests confirmed overfitting [27,28]. Variable Importance in Projection (VIP) values from OPLS-DA were used as a measure of metabolite importance. Univariate analysis (paired samples *t*-test and independent samples *t*-test) identified differentially abundant metabolites. False Discovery Rate (FDR) estimation was used to adjust for multiple comparisons [29]. Differential fold change (FC) was used to quantify metabolite abundance differences. The criteria for selecting differentially regulated metabolites were as follows: VIP ≥ 1, FC ≥ 1.5 or ≤ 0.75, and FDR < 0.05. Selected metabolites were used for further analysis.

## 3. Results

### 3.1. Anthropometric and Physiological Parameters

The results are displayed in Table 2. As shown, we observed no significant between-group differences from pre- to post-intervention. 

Within the PEP group, increases were observed in weight (2.28%), BMI (2.24%), body fat percentage (56.27%), and pulmonary function (13.76%). Within the PRO group, increases were seen in body fat percentage (25.13%) and pulmonary function (5.84%) from baseline to post-intervention. The change from baseline was greater for increases in body fat percentage (PEP: 56.27% vs. PRO: 25.13%) and pulmonary function (PEP: 13.76% vs. PRO: 5.84%) in the PEP group than in the PRO group.

### 3.2. Athletic Performance

#### 3.2.1. Aerobic Capacity

The results of the *t*-tests are displayed in Table 3. No significant differences were observed at baseline between the groups in any of the aerobic capacity indices. Post-intervention, blood glucose immediately after exhaustion was significantly lower in PEP than in PRO (PEP: 5.34 ± 0.5 vs. PRO: 6.36 ± 1.05), as was the change from baseline (PEP: −23.27%, PRO: −11.70%).

Within PEP, time to complete a 10 km roller skating event decreased (−1.12%) and blood glucose immediately after exhaustion decreased significantly (−23.27%), while individual lactate threshold (10.91%) and RPE post-exhaustion (7.41%) increased significantly. Within PRO, blood glucose at rest and immediately post-exhaustion decreased significantly from baseline after intervention (−18.34%; −11.70%). The change from baseline in blood glucose immediately after exhaustion was greater in PEP than in PRO (PEP: −23.27% vs. PRO: −11.7%).

**Figure 2:**
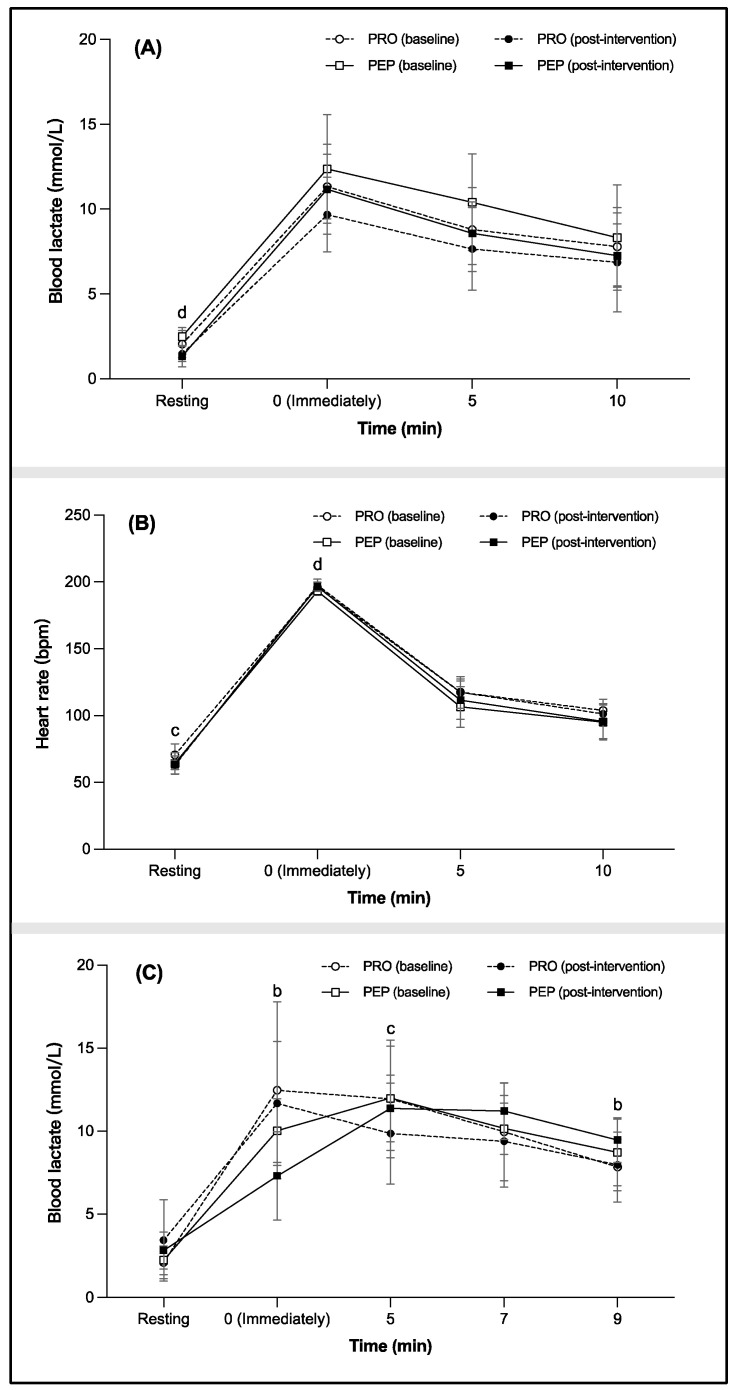
Comparative analysis of (**A**) blood lactate concentration levels, (**B**) heart rate during tests of aerobic capacity, and (**C**) blood lactate levels during tests of anaerobic capacity at baseline and following intervention for cross-country skiers supplemented with either wheat peptide bars or regular protein bars. Data are presented as mean ±SD. Significant differences between groups post-intervention (^b^ *p* < 0.05), within the PRO group (^c^ *p* < 0.05), and within the PEP group (^d^ *p* < 0.05).

The four (condition)-by-four (time) repeated-measures ANOVA results for blood lactate during aerobic capacity testing are displayed in Figure 2 and Supplementary Materials Table S5. No significant interaction effects or main effects of condition were observed, though a significant main effect of time was seen (*p* < 0.001, pη2 = 0.984). At rest, blood lactate post-intervention was significantly lower than baseline in PEP (−45.53%); both groups increased rapidly to peak levels immediately post-VO_2_max test exhaustion then declined over time. Blood lactate decreased significantly in PEP from immediately to 5 min post-exhaustion (11.17 ± 2.66 to 8.58 ± 1.85) and from 5 to 10 min post-exhaustion (8.58 ± 1.85 to 7.27 ± 1.86). Heart rate results showed a similar pattern, without interaction or condition effects, but a significant time effect was observed (*p* < 0.001, pη2 = 0.864), with heart rate at rest post-intervention lower than baseline in PRO (Δ = −9.99%). Heart rate increased rapidly to peak levels immediately post-exhaustion and then declined over time, with the PEP group higher than baseline immediately post-exhaustion (1.85%).

#### 3.2.2. Anaerobic Capacity and Strength

The results of the *t*-tests are displayed in Table 4. At baseline, resting blood glucose was significantly higher in PRO than in PEP (PRO: 6.76 ± 0.6 vs. PEP: 6.03 ± 0.69), with no other significant differences between groups for anaerobic capacity indices. Post-intervention, no significant differences existed between groups for any indices.

Within PEP, blood glucose immediately post-exercise decreased significantly from baseline (−13.13%). Within PRO, time to complete the 30 m sprint (−2.86%) and the hexagonal test (−3.3%) decreased significantly, and the fatigue index decreased significantly in both groups post-intervention (PRO: −28.37%; PEP: −29.88%). The change from baseline in blood glucose immediately post-exercise was greater in PEP than in PRO (PEP: −13.13% vs. PRO: 5.85%).

The results of the repeated-measures ANOVA (four conditions × five time points) for blood lactate during anaerobic exercise capacity testing are presented in Figure 2 and the Supplementary Materials Table S5. A significant interaction effect (*p* = 0.002, pη2 = 0.219) and a significant main effect of time (*p* < 0.001, pη2 = 0.984) were observed. At rest, there were no significant differences in blood lactate between the two groups or between the intervention and baseline measurements. Immediately post-exercise, blood lactate levels increased rapidly, but the PEP group showed significantly lower levels than the PRO group after the intervention (PEP: 7.31 ± 2.67 vs. PRO: 11.68 ± 3.74). Subsequently, the PRO group exhibited a continuous decrease in blood lactate over time, while the PEP group’s blood lactate continued to increase to its peak at 5 min post-exercise and then gradually decreased. Finally, at 9 min post-exercise, the PEP group showed significantly higher blood lactate levels than the PRO group after the intervention (PEP: 9.47 ± 1.34 vs. PRO: 7.98 ± 1.55).

### 3.3. Metabolomic Analysis

#### 3.3.1. Multivariate Analysis

**Figure 3:**
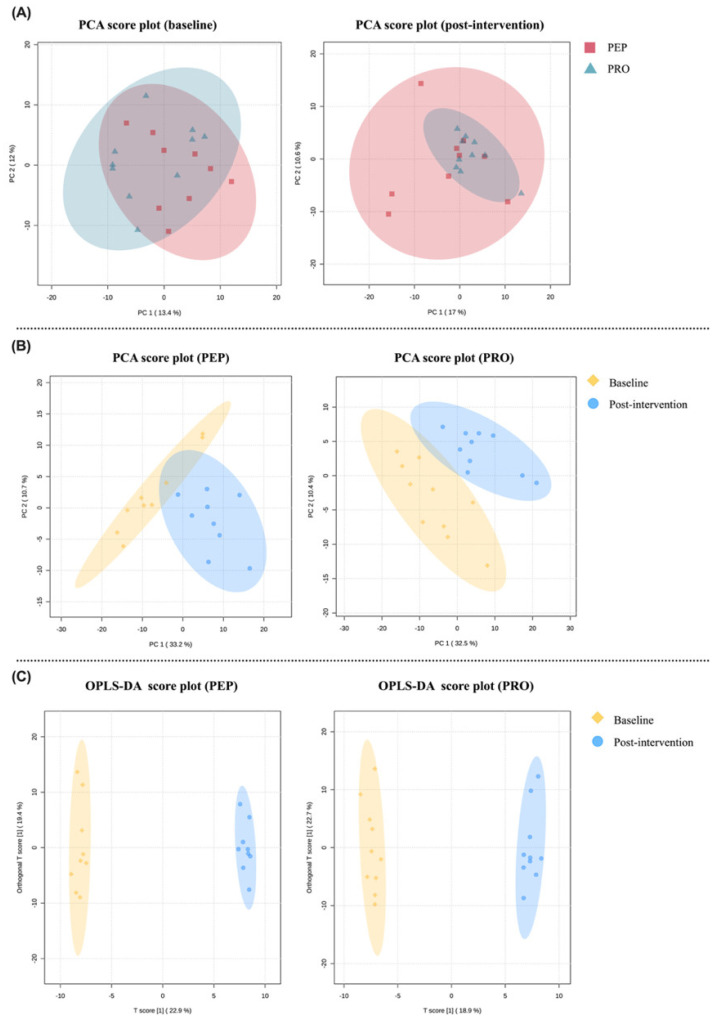
The PCA and OPLS-DA score plots. (**A**) PCA score plots compare the PEP and PRO groups at baseline (**left**) and post-intervention (**right**); (**B**) PCA score plots compare the different timepoints of baseline and post-intervention within the PEP group (**left**) and PRO group (**right**); (**C**) OPLS-DA score plots compare the different timepoints of baseline and post-intervention within the PEP group (**left**) and PRO group (**right**). The plots show the distribution of samples from the two groups based on principal component analysis (PCA) and/or Orthogonal Partial Least Squares Discriminant Analysis (OPLS-DA). Each point represents an individual sample, and the positioning of the points reflects their similarity or dissimilarity in terms of metabolite profiles.

Based on unsupervised PCA analysis, score plots of the first two principal components were generated for both groups at baseline and post-intervention (Figure 3A). However, no conclusion regarding clustering trends between groups could be drawn from these plots. Therefore, supervised OPLS-DA models were employed to amplify subtle differences, utilizing predictive and orthogonal principal components for improved separation of each cluster. While separation was observed between baselines, model evaluation parameters indicated insufficient prediction ability (R2Y = 0.809, Q2 = 0.193). A 100-response permutation test was performed at *p* < 0.05 to reduce overfitting in the OPLS-DA model [30]. Test parameters (R2Y = 0.994, *p* = 0.48; Q2 = 0.302, *p* = 0.17) showed overfitting issues, with R2Y representing model explanation of Y and Q2 representing prediction ability—values close to one indicate a more stable, reliable model. Conventionally, R2Y and Q2 should exceed 0.5 to ensure good prediction. Post-intervention OPLS-DA models between groups similarly showed insufficient prediction (R2Y = 0.718, Q2 = 0.158) and overfitting according to permutation testing (R2Y = 0.938, *p* = 0.72; Q2 = 0.177, *p* = 0.28).

However, scores plots based on within-group comparisons of the first two principal components (Figure 3B) showed some separation trends between metabolic profiles at baseline and post-intervention for the PEP and PRO groups. OPLS-DA models (Figure 3C) were thus applied to amplify subtle differences. Clearer clustering differentiation between baseline and post-intervention was observed for PEP (R2Y = 0.779, Q2 = 0.669) and PRO (R2Y = 0.717, Q2 = 0.6), with good prediction. After 100 permutations, models for PEP (R2Y = 0.997, *p* < 0.01; Q2 = 0.91, *p* < 0.01) and PRO (R2Y = 0.995, *p* < 0.01; Q2 = 0.84, *p* < 0.01) performed well without overfitting.

VIP values from OPLS-DA models were used to determine metabolite importance in constructed models, with VIP > 1 as the threshold for screening differential metabolites, analyzed further by univariate FC and FDR (see Supplementary Materials Table S6).

#### 3.3.2. Univariate Analysis

**Figure 4:**
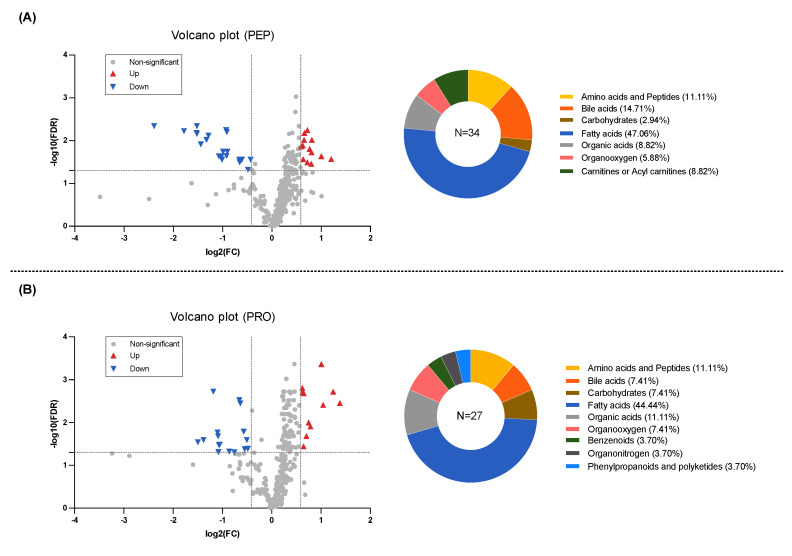
Volcano plots and differential metabolite classes in (**A**) PEP group; (**B**) PRO group. The x-axis represents log2(fold change), while the y-axis represents false discovery rate on a -log10 scale.

The PEP and PRO groups did not exhibit any significant differences in metabolites that met the predetermined criteria between the baseline and intervention measurements. However, based on the established criteria, the PEP group had a total of 32 important differentially abundant metabolites identified, with 13 metabolites upregulated and 21 metabolites downregulated (Figure 4A, Supplementary Materials Table S6). Among these metabolites, fatty acids accounted for the highest proportion, followed by bile acids and then amino acids and peptides, and the remaining metabolite categories included organic acids, carnitines or acyl carnitines, organooxygen, and carbohydrates.

For the PRO group, a total of 27 important differentially abundant metabolites were identified based on the established criteria, with 11 metabolites upregulated and 16 metabolites downregulated (Figure 4B, Supplementary Materials Table S6). Among these metabolites, fatty acids accounted for the highest proportion, followed by organic acids and then amino acids and peptides, and the remaining metabolite categories included bile acids, carbohydrates, organooxygen, benzenoids, organonitrogen compounds, and phenylpropanoids and polyketides.

#### 3.3.3. Functional Analysis of Metabolic Pathways

**Figure 5:**
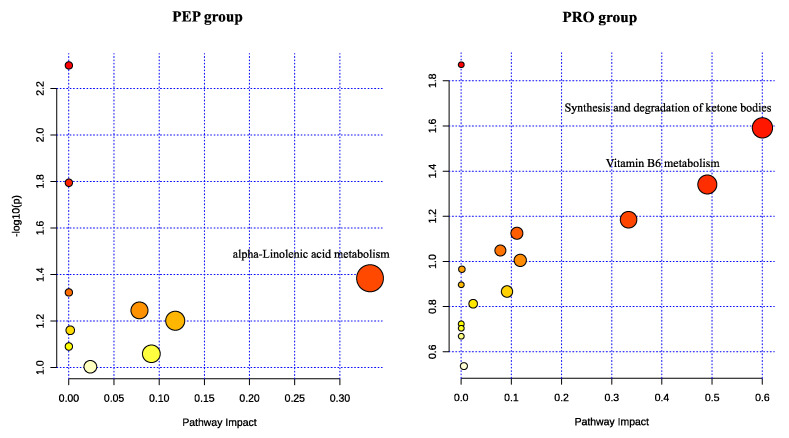
Pathway analysis overview of metabolic pathways in the PEP and PRO groups. The circles represent the involved pathways, and significantly changed pathways are labeled with names.

This study conducted a metabolic pathway analysis of differing metabolites based on the KEGG database. Pathways were considered significantly impacted when the impact > 0.1 and *p* < 0.05, and the results are shown in Figure 5. In the PEP group, with α-Linolenic acid metabolism exhibiting significant alteration. In the PRO group, 15 metabolic pathways were impacted, including the synthesis and degradation of ketone bodies and vitamin B6 metabolism.

## 4. Discussion

During a four-week trial period, 19 cross-country skiers were randomly assigned to receive either wheat peptides or regular proteins. The results of the study show that both groups demonstrated similar effects on their athletic performance, with some minor differences observed in specific aspects. Compared to baseline values, supplementation with wheat peptides resulted in significant increases in body fat percentage and pulmonary ventilation. Furthermore, the increase in lung capacity was significantly higher in the PEP group than in the PRO group. Additionally, during aerobic capacity testing, time to complete the 10 km roller skating even decreased markedly, as did individual lactate thresholds. However, supplementation with proteins did not elicit similar changes. Conversely, proteins supplementation significantly enhanced 30 m sprint and hexagon test performance among skiers undergoing anaerobic capacity testing.

In the aerobic performance assessment, we observed a notable decrease in resting blood glucose levels, which was either statistically significant or trending towards significance, after supplementation with wheat peptides or regular proteins compared to the baseline measurements. Typically, during rest to low-intensity exercise (25% VO_2_max), ATP production primarily relies on fatty acid oxidation [31], with plasma fatty acids serving as the main energy source, supplemented by a minor contribution from blood glucose [32]. The PRO group demonstrated a significant reduction in resting blood glucose levels, while the PEP group exhibited a non-significant decrease. It is worth noting that reduced resting blood glucose levels may indicate enhanced utilization of fat oxidation and an improved recovery response during low-intensity exercise [32]. Individuals with lower endurance capacity, both among athletes and within the general population, may experience elevated blood glucose concentrations during low-intensity exercise [32]. By activating enzymes involved in fatty acid oxidation, endurance athletes can enhance both their endurance and recovery capacities [32]. Furthermore, we observed a significant decrease in resting heart rate among the athletes following the consumption of regular proteins, suggesting an increased heart rate reserve and a more energy-efficient state during rest, potentially leading to improved endurance performance.

We also observed lower post-exercise blood glucose levels among athletes supplemented with wheat peptides than among those receiving regular proteins, during both aerobic and anaerobic capacity testing. In the PEP group, we observed a significant decrease in immediate post-exercise blood glucose levels compared to baseline levels, which further declined compared to resting conditions. This aligns with the physiological phenomenon of increased muscular uptake and utilization of glucose during exertion, thereby reducing its blood concentration [33]. However, we found that regular protein supplementation better stabilized blood glucose, preventing exercise-induced declines in its concentration.

In the aerobic performance test, athletes who were supplemented with wheat peptides exhibited a significant decrease in resting blood lactate levels compared to the baseline measurements. Previous research has indicated that during the low-intensity exercise phase, where total fat oxidation predominates, blood lactate concentrations appear to gradually decrease [31,34]. Recent studies have also highlighted the reduction in blood lactate as a relevant signal for recovery capacity in well-trained athletes during intermittent and endurance exercises [32]. Therefore, the lower blood lactate levels at rest may serve as an indicator of enhanced endurance performance and improved recovery capacity.

The variations in blood lactate levels during anaerobic capacity tests displayed different patterns. The PEP group exhibited significantly lower blood lactate levels immediately following exercise than the PRO group, suggesting that PEP intervention may assist in reducing lactate accumulation after anaerobic exercise. However, blood lactate levels in the PEP group continued to rise 5 min after exercise before gradually decreasing, whereas levels in the PRO group gradually reduced over time. This potentially implies a longer recovery time for the PEP group along with lower lactate clearance ability. 

**Figure 6:**
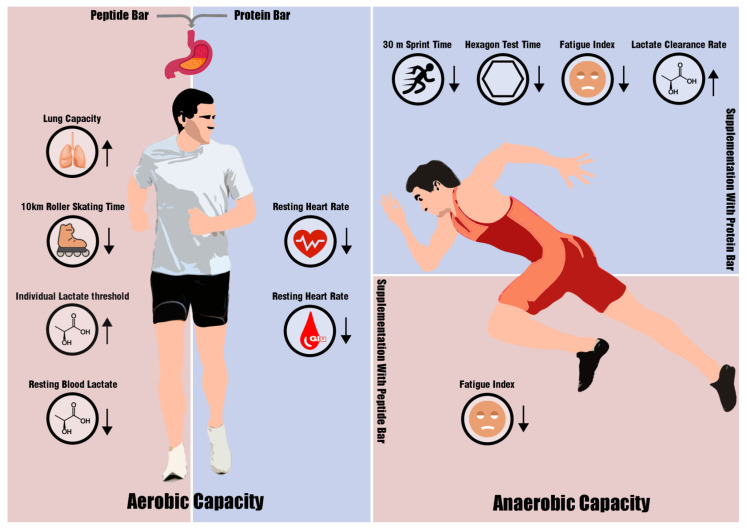
Illustration summarizing the described factors contributing to the improved aerobic and anaerobic capacities of cross-country skiers following supplementation with wheat peptide or regular protein bars.

Overall, the PEP group showed greater improvements in aerobic capacity, while the PRO group exhibited enhanced anaerobic capacity. A review by Cintineo et al. also reported that protein supplementation can benefit endurance or resistance performance in athletes [35]. Additionally, results from a recent meta-analysis found that co-ingestion of protein and carbohydrates during or after exercise exhibited additive effects on endurance performance relative to carbohydrates alone [36]. These studies support the conclusions of our study, i.e., that supplementary wheat peptides and regular proteins both provided beneficial effects on the fitness of cross-country skiers. However, we only observed differences in the enhancement of aerobic and anaerobic capacities between wheat peptides and conventional protein through intra-group changes. No significant differences were found in direct comparisons between the two groups, which aligns with the conclusions of the review we referenced earlier [25]. Hence, in further studies, it may be necessary to consider longer intervention durations or increased wheat peptide quantities to potentially discover efficacy advantages of wheat peptides relative to regular proteins in supplementation. A summary of the observed enhancement in athletic performance following supplementation with the wheat peptides or regular proteins is depicted in Figure 6.

We conducted high-throughput targeted metabolomics analysis on athletes’ serum samples. When the PCA analysis did not show clear clustering trends, we used an OPLS-DA model for supervised learning. However, the model’s predictive ability was insufficient, and overfitting issues were identified. Nevertheless, within-group comparisons revealed separation trends between baseline and post-intervention samples in both groups, which were further emphasized by the OPLS-DA model with good predictive performance. Univariate analysis identified differential metabolites in both the PEP and PRO groups, primarily involving fatty acids, bile acids, amino acids, and peptides.

These metabolomic results not only validate our previous findings on athletic performance but also provide insights into the metabolic improvements resulting from bar supplementation. Furthermore, there were differences in the categories of differential metabolites between the PEP group and the PRO group. Specifically, the α-linolenic acid metabolism pathway was notably altered in the PEP group, while the synthesis and degradation of ketone bodies and vitamin B6 metabolism showed significant changes in the PRO group.

α-Linolenic acid is an essential fatty acid belonging to the ω-3 series. Due to limited endogenous synthesis capacity in the human body [37], it is crucial to obtain sufficient α-linolenic acid through dietary intake [38]. Adequate intake of α-linolenic acid as part of a balanced diet may have multiple health benefits. Studies have shown an association between α-linolenic acid and reduced cardiovascular disease risk, possibly through mechanisms such as lowering blood triglyceride levels, reducing inflammation, improving endothelial function, and lowering blood pressure [39]. The metabolomics analysis revealed a reduction in α-linolenic acid following wheat peptide supplementation. This may be a contributing factor to the lower efficiency of lactate clearance in the PEP group compared to the PRO group, as α-linolenic acid is linked to hemorheology. No other adverse effects of downregulating α-linolenic acid metabolic pathways on exercise performance were observed. Therefore, specific biological connections and interactions may require further in-depth research for clarification.

In our study, we observed that the upregulation of acetoacetate after supplementation with regular proteins was closely related to changes in metabolism of ketone bodies. Acetoacetate is a precursor of ketone bodies, together with 3-hydroxybutyrate ester and acetone, which form ketone bodies [40]. The circulating levels of ketone bodies are determined by the rates of their production (ketogenesis) and utilization (ketolysis) [40]. They can reduce dependence on glucose, thereby preserving endogenous glycogen stores [41]. By serving as alternative fuel substrates, the presence of ketone bodies can help reduce carbohydrate consumption, potentially resulting in performance benefits during endurance exercise [42]. Importantly, after consumption of the regular proteins, we observed an upregulation of ketone bodies, which promoted the utilization of fatty acids while reducing dependence on carbohydrates. This finding aligns with the concurrent decrease in resting blood glucose levels observed during our aerobic performance test.

Additionally, we observed that pyridoxal (PL) was upregulated after supplementation with the regular proteins, along with upregulation of the vitamin B6 metabolism pathway. The de novo biosynthesis of vitamin B6 occurs in microorganisms and plants, but it has been lost in animals [43], suggesting that the changes in this pathway are likely due to dietary or gut microbiota factors. Nuts are a rich source of vitamin B6, and our bars contained nut ingredients, which may explain the upregulation of vitamin B6 synthesis and metabolism. Pyridoxal 5′-phosphate (PLP) is the active form of vitamin B6 [44], synthesized from PL by PL kinase [45]. PLP acts as a coenzyme in the synthesis of glycine and hemoglobin [46]. Additionally, it binds to two sites on hemoglobin, enhancing its oxygen-binding capacity [47].The upregulation of PL in this pathway has beneficial physiological effects, potentially improving exercise performance through enhanced oxygen supply and other mechanisms. This further supports the observed decrease in resting heart rate after supplementation with regular proteins in our aerobic performance test, indicating a more energy-efficient state following improvements in oxygen uptake and transport capacity.

This study represents a pioneering investigation comparing the effects of wheat peptide and regular protein supplementation on endurance athletes’ performance capabilities, offering a novel and significant research pathway. Additionally, our evaluation of the impacts of wheat peptides on athletes’ blood metabolomes provides valuable insights into the potential roles of wheat peptides in human metabolism and their connection to performance. These findings contribute to the scientific rationale guiding endurance athletes in selecting appropriate energy supplements.

However, it is important to acknowledge certain limitations of this study. Firstly, the sample size was limited due to the inclusion of professional cross-country skiers, which may constrain the generalizability and statistical reliability of the findings. Expanding the sample size would enhance the reliability and applicability of the research. Secondly, the intervention duration was relatively short, potentially limiting the ability to clearly demonstrate differences in efficacy between the wheat peptides and regular proteins. Extending the intervention duration would yield more robust and comprehensive results. Lastly, the inclusion of athletes under the age of 18 introduces physiological and metabolic differences compared to adults. Caution should be exercised when interpreting the results to account for these age-related variations.

## 5. Conclusions

In summary, our study found that wheat oligopeptides did not provide a significant advantage over regular proteins in overall exercise performance for cross-country skiers. However, wheat peptides showed a more pronounced effect on enhancing aerobic capacity. The blood metabolite profile did not differ significantly between athletes who consumed the two types of bars, but specific metabolic pathways were affected differently: wheat peptides influenced α-linolenic acid, while proteins influenced synthesis and degradation of ketone bodies, as well as vitamin B6 metabolism. Athletes may consider both types of energy supplements, with wheat peptides potentially being more suitable for improving aerobic capacity. Individual variations and specific metabolic requirements should be considered when selecting the most appropriate energy supplement.

## Figures and Tables

**Table 1:** Participant characteristics (mean ± SD).

Variable	PRO Group (*n* = 10)	PEP Group (*n* = 9)	*p*-Value
Age (years)	16.8 ± 1.32	16.78 ± 1.3	0.971
Height (cm)	175.6 ± 4.97	176.67 ± 5.61	0.666
Weight (kg)	63.77 ± 7.92	61.33 ± 4.47	0.428
Training experience (years)	3.20 ± 1.03	3.17 ± 1.27	0.743
BMI (kg/m2)	20.53 ± 1.56	19.48 ± 1.04	0.106
Body fat (%)	7.99 ± 2.64	6.51 ± 2.31	0.213
1500 m run (s)	280.2 ± 14.76	286.44 ± 14.12	0.171
VO_2_max (mL/kg/min)	64 ± 3.4	62.72 ± 2.89	0.136
30 m sprint (s)	4.28 ± 0.28	4.18 ± 0.12	0.537
Peak power (W/kg)	10.67 ± 1.46	10.96 ± 0.76	0.661

BMI = body mass index.

**Table 2:** Selected anthropometric and physiological parameters of cross-country skiers in the PEP and PRO groups (mean ± SD).

Variable	PRO Group (*n* = 10)			PEP Group (*n* = 9)		
	Baseline	Post-Intervention	Δ	Baseline	Post-Intervention	Δ
Body weight (kg)	63.77 ± 7.92	64.43 ± 7.93	1.10%	61.33 ± 4.47	62.76 ± 4.96 ***	2.28%
BMI(kg/m^2^)	20.53 ± 1.56	20.68 ± 1.63	0.74%	19.48 ± 1.04	19.92 ± 1.23 ***	2.24%
Body fat (%)	7.99 ± 2.64	9.4 ± 1.69 *	25.13%	6.51 ± 2.31	9.77 ± 2.57 ***	56.27% †
Skeletal muscle mass (kg)	33.13 ± 4.29	33.06 ± 4.03	−0.08%	32.33 ± 2.72	32.08 ± 2.95	−0.85%
Lung capacity (L)	4.6 ± 0.65	4.84 ± 0.51 **	5.84%	4.21 ± 0.56	4.77 ± 0.49 ***	13.76% †

Δ represents the rate of change relative to the baseline after the intervention; BMI = body mass index. Significant difference within group (* *p* < 0.05, ** *p* < 0.01, *** *p* < 0.001); significant differences in Δ between groups († *p* < 0.05).

**Table 3:** Selected aerobic capacity indicators of cross-country skiers in PEP and PRO groups (mean ± SD).

Variable	PRO Group (*n* = 10)			PEP Group (*n* = 9)		
	Baseline	Post-Intervention	Δ	Baseline	Post-Intervention	Δ
1500 m run (s)	280.2 ± 14.76	285.1 ± 15.68	1.94%	289.67 ± 14.02	286.44 ± 14.12	−0.94%
10 km roller skating (s)	1607.1 ± 99.54	1602.2 ± 100.32	−0.30%	1608.89 ±117.71	1590.44 ±111.85 *	−1.12%
VO2max (mL/kg/min)	64 ± 3.4	63.86 ± 3.53	−0.16%	61.56 ± 3.4	62.72 ± 2.89	2%
Lactate threshold	3.12 ± 0.4	3.13 ± 0.54	0.23%	2.81 ± 0.44	3.1 ± 0.42	11.77%
Individual lactate threshold	49.4 ± 3.6	50 ± 6.63	1.04%	46.11 ± 6.45	50.57 ± 5.69 *	10.91%
RPE post-exhaustion	16.5 ± 1.43	16.9 ± 1.1	3.29%	15.78 ± 0.97	16.89 ± 0.6 *	7.41%
Blood glucose (mmol/L)						
Resting	6.6 ± 0.49	5.37 ± 0.55 ***	−18.34%	6.09 ± 1.02	5.34 ± 0.59	−9.03%
Immediately ^a^	7.17 ± 0.56	6.36 ± 1.05 **	−11.70%	7.09 ± 1.07	5.34 ± 0.5 ** #	−23.27% †

Δ represents the rate of change relative to the baseline after the intervention; RPE = rate of perceived exertion; ^a^ represents post-exhaustion. Significant differences within groups (* *p* < 0.05, ** *p* < 0.01, *** *p* < 0.001); significant differences between groups (# *p* < 0.05); significant differences in Δ between groups († *p* < 0.05).

**Table 4:** Selected anaerobic capacity and strength indicators of cross-country skiers in PEP and PRO groups (mean ± SD).

Variable	PRO Group (*n* = 10)			PEP Group (*n* = 9)		
	Baseline	Post-Intervention	Δ	Baseline	Post-Intervention	Δ
30 m sprint (s)	4.28 ± 0.28	4.15 ± 0.21 *	−2.86%	4.21 ± 0.15	4.18 ± 0.12	−0.81%
Hexagon test of agility (s)	9.12 ± 0.48	8.81 ± 0.41 *	−3.30%	9.05 ± 0.38	8.83 ± 0.37	−2.33%
Bench press relative strength	1.13 ± 0.2	1.1 ± 0.16	−1.59%	1.05 ± 0.22	1.01 ± 0.18	−2.33%
CMJ (m)	0.38 ± 0.06	0.34 ± 0.04	−10.25%	0.37 ± 0.07	0.34 ± 0.07	−4.14%
SJ (m)	0.32 ± 0.05	0.3 ± 0.05	−5.13%	0.29 ± 0.07	0.3 ± 0.05	8.01%
Average power (W/kg)	8.58 ± 0.83	8.67 ± 0.73	1.29%	8.35 ± 0.85	8.7 ± 0.42	4.87%
Peak power (W/kg)	10.67 ± 1.46	10.77 ± 1.39	1.15%	10.4 ± 1.11	10.96 ± 0.76	6.31%
Fatigue index	0.28 ± 0.08	0.19 ± 0.05 *	−28.37%	0.29 ± 0.06	0.2 ± 0.04 ***	−29.88%
Blood glucose (mmol/L)						
Resting	6.76 ± 0.6	6.48 ± 0.8	−3.34%	6.03 ± 0.69 #	6.32 ± 0.94	5.15%
Immediately ^b^	6.29 ± 0.4	6.64 ± 1.23	5.85%	6.67 ± 0.66	5.76 ± 0.59 **	−13.13% †

Δ represents the rate of change relative to the baseline after the intervention; CMJ = countermovement jump; SJ = squat jump; ^b^ represents post-exercise. Significant differences within groups (* *p* < 0.05, ** *p* < 0.01, *** *p* < 0.001); significant differences between groups (# *p* < 0.05); significant differences in Δ between groups († *p* < 0.05)

## Data Availability

All data generated or analyzed during this study are included within the article or are provided in the Supplementary Materials.

## Supplementary Materials

The following supporting information can be downloaded at: https://www.mdpi.com/article/10.3390/metabo14100538/s1, Table S1: Daily macronutrient intake and total energy intake; Table S2: Intervention-period weekly training plan for the cross-country skiers; Table S3: Selected athletic performance indicators of cross-country skiers in the PEP and PRO groups; Table S4: Differential metabolites screened according to predetermined criteria; Table S5: Selected athletic performance indicators of cross-country skiers in PEP and PRO groups (Mean ± SD); Table S6: Differential metabolites screened according to predetermined criteria.

## References

[1] Holmberg H.-C. (2015). The elite cross-country skier provides unique insights into human exercise physiology. Scand. J. Med. Sci. Sports.

[2] Sandbakk Ø., Holmberg H.-C. (2017). Physiological Capacity and Training Routines of Elite Cross-Country Skiers: Approaching the Upper Limits of Human Endurance. Int. J. Sports Physiol. Perform..

[3] Hébert-Losier K., Zinner C., Platt S., Stöggl T., Holmberg H.C. (2017). Factors that Influence the Performance of Elite Sprint Cross-Country Skiers. Sports Med..

[4] Losnegard T. (2019). Energy system contribution during competitive cross-country skiing. Eur. J. Appl. Physiol..

[5] Sandbakk Ø., Holmberg H.-C., Leirdal S., Ettema G. (2010). Metabolic rate and gross efficiency at high work rates in world class and national level sprint skiers. Eur. J. Appl. Physiol..

[6] Rusko H. (2008). The Handbooks of Sports Medicine and Science: Cross Country Skiing.

[7] Heikura I.A., Kettunen O., Garthe I., Holmlund H., Sandbakk S.B., Valtonen M., Ihalainen J.K. (2021). Energetic demands and nutritional strategies of elite cross-country skiers during tour de ski: A narrative review. J. Sci. Sport Exerc..

[8] Tarnopolsky M. (2004). Protein Requirements for Endurance Athletes. Eur. J. Sport Sci..

[9] Moore D.R. (2015). Nutrition to Support Recovery from Endurance Exercise: Optimal Carbohydrate and Protein Replacement. Curr. Sports Med. Rep..

[10] Moore D.R., Camera D.M., Areta J.L., Hawley J.A. (2014). Beyond muscle hypertrophy: Why dietary protein is important for endurance athletes. Appl. Physiol. Nutr. Metab..

[11] Lin Y.-N., Tseng T.-T., Knuiman P., Chan W.P., Wu S.-H., Tsai C.-L., Hsu C.-Y. (2021). Protein supplementation increases adaptations to endurance training: A systematic review and meta-analysis. Clin. Nutr..

[12] Matthews D.M., Adibi S.A. (1976). Peptide Absorption. Gastroenterology.

[13] Addison J.M., Burston D., Matthews D. (1972). Evidence for active transport of the dipeptide glycylsarcosine by hamster jejunum in vitro. Clin. Sci..

[14] Gardner M. (1975). Absorption of amino acids and peptides from a complex mixture in the isolated small intestine of the rat. J. Physiol..

[15] Tagari H., Webb K., Theurer B., Huber T., De Young D., Cuneo P., Santos J., Simas J., Sadik M., Alio A. (2008). Mammary uptake, portal-drained visceral flux, and hepatic metabolism of free and peptide-bound amino acids in cows fed steam-flaked or dry-rolled sorghum grain diets. J. Dairy Sci..

[16] Kitakaze T., Sakamoto T., Kitano T., Inoue N., Sugihara F., Harada N., Yamaji R. (2016). The collagen derived dipeptide hydroxyprolyl-glycine promotes C2C12 myoblast differentiation and myotube hypertrophy. Biochem. Biophys. Res. Commun..

[17] Zdzieblik D., Oesser S., Baumstark M.W., Gollhofer A., König D. (2015). Collagen peptide supplementation in combination with resistance training improves body composition and increases muscle strength in elderly sarcopenic men: A randomised controlled trial. Br. J. Nutr..

[18] Clifford T., Ventress M., Allerton D.M., Stansfield S., Tang J.C.Y., Fraser W.D., Vanhoecke B., Prawitt J., Stevenson E. (2019). The effects of collagen peptides on muscle damage, inflammation and bone turnover following exercise: A randomized, controlled trial. Amino Acids.

[19] Hansen M., Bangsbo J., Jensen J., Bibby B.M., Madsen K. (2015). Effect of Whey Protein Hydrolysate on Performance and Recovery of Top-Class Orienteering Runners. Int. J. Sport Nutr. Exerc. Metab..

[20] Jendricke P., Kohl J., Centner C., Gollhofer A., König D. (2020). Influence of Specific Collagen Peptides and Concurrent Training on Cardiometabolic Parameters and Performance Indices in Women: A Randomized Controlled Trial. Front. Nutr..

[21] Oosthuyse T., Carstens M., Millen A.M.E. (2015). Whey or Casein Hydrolysate with Carbohydrate for Metabolism and Performance in Cycling. Int. J. Sports Med..

[22] Lollo P.C.B., Amaya-Farfan J., Faria I.C., Salgado J.V.V., Chacon-Mikahil M.P.T., Cruz A.G., Oliveira C.A.F., Montagner P.C., Arruda M. (2014). Hydrolysed whey protein reduces muscle damage markers in Brazilian elite soccer players compared with whey protein and maltodextrin. A twelve-week in-championship intervention. Int. Dairy J..

[23] Qu Y., Ji H., Song W., Peng S., Zhan S., Wei L., Chen M., Zhang D., Liu S. (2022). The anti-fatigue effect of the Auxis thazard oligopeptide via modulation of the AMPK/PGC-1α pathway in mice. Food Funct..

[24] Zheng Z.-Q., Geng Z.-H., Liu J.-X., Guo S.-T. (2017). Compressed food with added functional oligopeptides improves performance during military endurance training. Asia Pac. J. Clin. Nutr..

[25] Morgan P.T., Breen L. (2021). The role of protein hydrolysates for exercise-induced skeletal muscle recovery and adaptation: A current perspective. Nutr. Metab..

[26] Cohen J. (2013). Statistical Power Analysis for the Behavioral Sciences.

[27] Worley B., Powers R. (2013). Multivariate analysis in metabolomics. Curr. Metabolomics.

[28] Worley B., Powers R. (2016). PCA as a practical indicator of OPLS-DA model reliability. Curr. Metabolomics.

[29] Chen Y., Li E.-M., Xu L.-Y. (2022). Guide to Metabolomics Analysis: A Bioinformatics Workflow. Metabolites.

[30] Szymańska E., Saccenti E., Smilde A.K., Westerhuis J.A. (2012). Double-check: Validation of diagnostic statistics for PLS-DA models in metabolomics studies. Metabolomics.

[31] Philp A., Macdonald A.L., Watt P.W. (2005). Lactate–a signal coordinating cell and systemic function. J. Exp. Biol..

[32] Yang W.-H., Park H., Grau M., Heine O. (2020). Decreased Blood Glucose and Lactate: Is a Useful Indicator of Recovery Ability in Athletes?. Int. J. Environ. Res. Public Health.

[33] Colberg S.R., Hernandez M.J., Shahzad F. (2013). Blood glucose responses to type, intensity, duration, and timing of exercise. Diabetes Care.

[34] Beneke R., Leithäuser R.M., Ochentel O. (2011). Blood lactate diagnostics in exercise testing and training. Int. J. Sports Physiol. Perform..

[35] Cintineo H.P., Arent M.A., Antonio J., Arent S.M. (2018). Effects of protein supplementation on performance and recovery in resistance and endurance training. Front. Nutr..

[36] Kloby Nielsen L.L., Tandrup Lambert M.N., Jeppesen P.B. (2020). The effect of ingesting carbohydrate and proteins on athletic performance: A systematic review and meta-analysis of randomized controlled trials. Nutrients.

[37] Hussein N., Ah-Sing E., Wilkinson P., Leach C., Griffin B.A., Millward D.J. (2005). Long-chain conversion of [13C]linoleic acid and alpha-linolenic acid in response to marked changes in their dietary intake in men. J. Lipid Res..

[38] Zárate R., el Jaber-Vazdekis N., Tejera N., Pérez J.A., Rodríguez C. (2017). Significance of long chain polyunsaturated fatty acids in human health. Clin. Transl. Med..

[39] Burdge G. (2006). Metabolism of α-linolenic acid in humans. Prostaglandins Leukot. Essent. Fat. Acids.

[40] Fukao T., Lopaschuk G.D., Mitchell G.A. (2004). Pathways and control of ketone body metabolism: On the fringe of lipid biochemistry. Prostaglandins Leukot. Essent. Fat. Acids.

[41] Cox P.J., Clarke K. (2014). Acute nutritional ketosis: Implications for exercise performance and metabolism. Extrem. Physiol. Med..

[42] Sansone M., Sansone A., Borrione P., Romanelli F., Di Luigi L., Sgrò P. (2018). Effects of ketone bodies on endurance exercise. Curr. Sports Med. Rep..

[43] Tambasco-Studart M., Titiz O., Raschle T., Forster G., Amrhein N., Fitzpatrick T.B. (2005). Vitamin B6 biosynthesis in higher plants. Proc. Natl. Acad. Sci..

[44] Ramos R.J., Albersen M., Vringer E., Bosma M., Zwakenberg S., Zwartkruis F., Jans J.J., Verhoeven-Duif N.M. (2019). Discovery of pyridoxal reductase activity as part of human vitamin B6 metabolism. Biochim. Et Biophys. Acta (BBA)-Gen. Subj..

[45] Hanna M.C., Turner A.J., Kirkness E.F. (1997). Human pyridoxal kinase: cDNA cloning, expression, and modulation by ligands of the benzodiazepine receptor. J. Biol. Chem..

[46] Parra M., Stahl S., Hellmann H. (2018). Vitamin B₆ and Its Role in Cell Metabolism and Physiology. Cells.

[47] Combs G.F., McClung J.P. (2016). The Vitamins: Fundamental Aspects in Nutrition and Health.

