# Supplementary material for: Wheat Peptides as Catalysts for Athletic Performance Improvement in Cross-Country Skiers: A Randomized Controlled Trial
Source: Metabolites. 2024 Oct 7;14(10):538. doi: 10.3390/metabo14100538 (PMC11509286; doi:10.3390/metabo14100538)
Supplement: Supplementary file 1 [file metabolites-14-00538-s001.zip › metabolites-3162109-supplementary.pdf]

Table S1. Daily macronutrient intake and total energy intake (Mean ± SD)

Time	Dietary Indicator	PRO Group (n=10)	PEP Group (n=9)	p-value
**Week 1**	Protein (g)	113.09±15.38	115.02±19.73	0.741
	Fat (g)	107.44±23.58	101.39±15.25	0.685
	Carbohydrate (g)	569.01±125.03	603.36±121.73	0.468
	Energy intake (kcal)	3653.14±389.03	3407.71±627.84	0.447
**Week 2**	Protein (g)	114.24±16.64	109.77±13.43	0.773
	Fat (g)	105.26±27.14	107.29±16.25	0.655
	Carbohydrate (g)	590.84±189.32	601.88±193.23	0.186
	Energy intake (kcal)	3487.55±713.85	3601.88±103.81	0.469
**Week 3**	Protein (g)	111.59±17.28	107.10±38.84	0.552
	Fat (g)	106.59±27.35	111.61±14.83	0.338
	Carbohydrate (g)	578.89±145.03	604.98±147.61	0.289
	Energy intake (kcal)	3227.15±697.83	3457.38±977.79	0.512
**Week 4**	Protein (g)	114.46±24.03	109.87±15.38	0.354
	Fat (g)	105.82±27.95	114.82±35.46	0.256
	Carbohydrate (g)	601.07±211.29	589.29±117.06	0.345
	Energy intake (kcal)	3579.65±294.01	3305.17±909.21	0.624

Table S2. Training Time and Heart Rate During the Training Period (Mean ± SD)

Time	Monitoring Indicator	PRO Group (n=10)	PEP Group (n=9)	p-value
**Week 1**	Training Duration (hours)	22.32±2.31	21.47±3.46	0.218
	Training Heart Rate (bpm)	151.61±11.17	147.28±13.72	0.652
**Week 2**	Training Duration (hours)	23.21±2.12	22.88±3.17	0.436
	Training Heart Rate (bpm)	146.35±9.69	148.46±8.36	0.362
**Week 3**	Training Duration (hours)	20.16±2.14	21.21±3.05	0.281
	Training Heart Rate (bpm)	147.38±6.19	149.16±5.63	0.461
**Week 4**	Training Duration (hours)	22.78±3.55	22.37±2.62	0.172
	Training Heart Rate (bpm)	148.15±5.38	147.16±5.67	0.321

Table S3. Sleep and Blood Pressure Monitoring (Mean ± SD)

Time	Indicator	PRO Group (n=10)	PEP Group (n=9)	p-value
	Sleep Duration (hours)	58.64±4.56	59.52±6.31	0.358
	Deep Sleep (hours)	59.36±5.54	57.98±4.67	0.541
**Week 1**	Systolic Blood Pressure （mmHg）	118.25±4.13	116.12±3.69	0.321
	Diastolic Blood Pressure （mmHg）	69.39±6.25	68.17±6.21	0.584
	Sleep Heart Rate (bpm)	57.18±3.15	58.29±2.86	0.564
	Sleep Duration (hours)	59.54±7.26	56.89±9.14	0.416
	Deep Sleep (hours)	58.36±7.54	56.38±6.67	0.711
**Week 2**	Systolic Blood Pressure （mmHg）	117.55±6.33	118.28±6.49	0.256
	Diastolic Blood Pressure （mmHg）	67.19±6.25	69.48±3.29	0.634
	Sleep Heart Rate (bpm)	59.16±4.25	59.19±6.87	0.267
	Sleep Duration (hours)	58.21±4.06	57.25±6.24	0.186
	Deep Sleep (hours)	57.26±6.51	59.18±5.34	0.658
**Week 3**	Systolic Blood Pressure （mmHg）	118.31±8.13	119.28±9.19	0.656
	Diastolic Blood Pressure （mmHg）	68.26±5.34	68.48±5.09	0.341
	Sleep Heart Rate (bpm)	56.66±3.25	57.19±3.07	0.726
	Sleep Duration (hours)	57.38±3.16	59.19±5.24	0.614
	Deep Sleep (hours)	59.06±6.54	58.38±3.47	0.127
**Week 4**	Systolic Blood Pressure （mmHg）	119.15±3.13	117.08±6.09	0.562
	Diastolic Blood Pressure （mmHg）	69.29±4.21	69.22±3.19	0.434
	Sleep Heart Rate (bpm)	58.19±3.25	58.67±4.87	0.621

Table S4. Intervention period weekly training plan for the cross-country skiers

Week 1		
**Date**	**Morning**	**Afternoon**
Monday, Oct 10th	Traditional Skiing: Warm-up 20 min / Large laps for men: 20 laps x 3 / Cool-down 30 min	"Integrated Strength: Jog 20 min, Gymnastics 10 min / Circuit strength training / Six exercises / 60 min. End activity: Stretching and relaxation 30 min."
Tuesday, Oct 11th	Freestyle Skiing: Warm-up 15 min / Technical sliding: 80 min / Cool-down 30 min	Aerobic Endurance: Warm-up 10 min / 20km / Cool-down 30 min
Wednesday, Oct 12th	Traditional Skiing: Warm-up 15 min / Timed small laps: 60 laps / Cool-down 30 min	General Integrated Training: Jog 30 min, Gymnastics 10 min / (Agility, Balance, Imitation) 60 min / Stretching and relaxation 30 min
Thursday, Oct 13th	Traditional Skiing: Warm-up 20 min / Large laps: 20 laps x 3 / Cool-down 30 min	Specific Strength Training: Jog 20 min, Gymnastics 10 min / Specific lower limb strength 60 min / End activity 30 min
Friday, Oct 14th	Freestyle Skiing: Warm-up 15 min / Technical sliding: 80 min / Cool-down 30 min	Specific Strength Training: Warm-up 10 min / Aerobic pole-assisted imitation running 80 min / Cool-down 30 min
Saturday, Oct 15th	Freestyle Skiing: Warm-up 15 min / Timed small laps: 60 laps / Cool-down 30 min	Rest and Adjustment
Sunday, Oct 16th	Specific Strength Training: Warm-up 10 min / Pole-assisted uphill climb / Flexibility and relaxation activities 30 min	Rest and Adjustment
**Week 2**		
**Date**	**Morning**	**Afternoon**

Monday, Oct 17	Ski pursuit training: 15 min warm-up / traditional large circle 20 laps + freestyle large circle 20 laps / 30 min cool-down	Preparation: 20 min jog, 10 min gymnastics: Comprehensive strength: Pull-ups for 5 min + Sit-ups for 60 sec + Arm and leg linkage for 60 sec + Left side support for 60 sec + Right side support for 60 sec + Box jumps for 60 sec + Ski machine for 5 min / 5 sets / Intervals of 3 min / Cool-down 30 min
Tuesday, Oct 18	Traditional short-distance skiing: 15 min warm-up / 5 large laps x 5 / 30 min cool-down	Aerobic endurance: 10 min warm-up / 10 km (Requirement: under 4'00'' per km) Imitation practice for 30 min / 30 min cool-down
Wednesday, Oct 19	Ski pursuit training: 15 min warm-up / traditional large circle 20 laps + freestyle large circle 20 laps / 30 min cool-down	Rest and adjustment
Thursday, Oct 20	Freestyle short-distance skiing: 15 min warm-up / 5 large laps x 5 / 30 min cool-down	Aerobic endurance: 10 min warm-up / 10 km / 20 min gymnastics / Lower body specific strength training: Resistance band lateral kicks with partners 50m + Imitation uphill running 80m (8 sets) / 30 min cool-down
Friday, Oct 21	Traditional skiing: 15 min warm-up / Aerobic endurance technical skiing 40 laps / 30 min cool-down	Aerobic endurance: 10 min warm-up / 10 km / 20 min gymnastics / Upper body strength (Pull-ups 120-150 / Prone row / Bench press / Barbell press / Leg press) / 30 min cool-down
Saturday, Oct 22	Freestyle skiing: 15 min warm-up / Aerobic endurance technical skiing 40 laps / 30 min cool-down	Rest and adjustment
Sunday, Oct 23	Specific strength and endurance: 10 min warm-up / Pole-assisted imitation running for 90 min / 30 min cool-down	Rest and adjustment

Week 3

Date	Morning	Afternoon
Monday, Oct 24	Ski pursuit training: 15 min warm-up / traditional large circle 25 laps + freestyle large circle 25 laps / 30 min cool-down	Preparation: 20 min jog, 10 min gymnastics: High-intensity interval training with bodyweight (eight exercises) two continuous sets as one group / 4 groups / Relaxation 30 min
Tuesday, Oct 25	Traditional short-distance skiing: 15 min warm-up / 4 large laps x	Aerobic endurance: 10 min warm-up / 15 km (Requirement: between 4'00'' and 4'30''

	5 / Sliding down the big slope x 5 / 30 min cool-down	per km) / 30 min cool-down
Wednesday, Oct 26	Freestyle skiing: 15 min warm-up / timed laps small circle: 60 laps / 30 min cool-down	Rest and adjustment
Thursday, Oct 27	Ski pursuit training: 15 min warm-up / traditional large circle 25 laps + freestyle large circle 25 laps / 30 min cool-down	Preparation: 20 min jog, 10 min gymnastics: Specific upper body strength: Pull-ups for 10 min / Barbell bench press / Dumbbell raises / 3 sets / Cool-down 30 min
Friday, Oct 28	Freestyle short-distance skiing: 15 min warm-up / 4 large laps x 5 / Sliding down the big slope x 5 / 30 min cool-down	Specific strength training: 10 min warm-up / Aerobic pole-assisted imitation running for 80 min / 30 min cool-down
Saturday, Oct 29	Traditional skiing: 15 min warm-up / timed laps small circle: 60 laps / 30 min cool-down	Rest and adjustment
Sunday, Oct 30	Preparation: 20 min jog, 10 min gymnastics: Specific lower body strength: Flip tires 30m / Resistance band lateral kicks with partners 60m / Weighted lunges 100m / 5 sets / Cool-down with 30 min jog and 20 min stretching	Rest and adjustment

Week 4

Date	Morning	Afternoon
Monday, Oct 31	Ski pursuit training: 15 min warm-up / traditional large circle 25 laps + freestyle large circle 25 laps / 30 min cool-down	Preparation: 20 min jog, 10 min gymnastics: High-intensity interval training with bodyweight (eight exercises) two continuous sets as one group / 4 groups / Relaxation 30 min
Tuesday, Nov 1	Traditional short-distance skiing: 15 min warm-up / 4 large laps x 5 / Sliding down the big slope x 5 / 30 min cool-down	Aerobic endurance: 10 min warm-up / 15 km (Requirement: between 4'00'' and 4'30'' per km) / 30 min cool-down
Wednesday, Nov 2	Freestyle skiing: 15 min warm-up / timed laps small circle: 60 laps / 30 min cool-down	Rest and adjustment
Thursday, Nov 3	Ski pursuit training: 15 min warm-up / traditional large circle 25 laps + freestyle large circle 25 laps / 30 min cool-down	Preparation: 20 min jog, 10 min gymnastics: Specific upper body strength: Pull-ups for 10 min / Barbell bench press / Dumbbell raises / 3 sets / Cool-down 30 min

Friday, Nov 4	Freestyle short-distance skiing: 15 min warm-up / 4 large laps x 5 / Sliding down the big slope x 5 / 30 min cool-down	Specific strength training: 10 min warm-up / Aerobic pole-assisted imitation running for 80 min / 30 min cool-down
Saturday, Nov 5	Traditional skiing: 15 min warm-up / timed laps small circle: 60 laps / 30 min cool-down	Rest and adjustment
Sunday, Nov 6	Preparation: 20 min jog, 10 min gymnastics: Specific lower body strength: Flip tires 30m / Resistance band lateral kicks with partners 60m / Weighted lunges 100m / 5 sets / Cool-down with 30 min jog and 20 min stretching	Rest and adjustment

Table S5. Selected athletic performance indicators of cross-country skiers in PEP and PRO groups (Mean ± SD)

Variable	PRO group (n = 10)		PEP group (n = 9)		Two-way ANOVA P-value (pη 2)		
	Baseline	Post-intervention	Baseline	Post-intervention	Interaction	Time	Intervention
**Aerobic capacity**							
**Blood lactate (mmol/L)**					0.284 (0.1)	<0.001 (0.984)	0.094 (0.169)
**Resting**	2.05 ± 0.79	1.48 ± 0.46	2.48 ± 0.56	1.33 ± 0.64**			
0 (Immediately)[a]	11.33 ± 1.91†††	9.68 ± 2.19†††	12.37 ± 3.2†††	11.17 ± 2.66†††			
5 minutes[a]	8.8 ± 2.48†	7.66 ± 2.45	10.41 ± 2.85	8.58 ± 1.85†			
10 minutes[a]	7.79 ± 2.31	6.87 ± 2.91	8.32 ± 3.11†††	7.27 ± 1.86†			
**Heart rate (bpm)**					0.786 (0.047)	<0.001 (0.864)	0.107 (0.162)
Resting	70.5 ± 8.29	62.8 ± 3.23**	64.22 ± 7.97	63.11 ± 6.9			

0 (Immediately)[a]	195.9 ± 3.99†††	197.5 ± 4.65†††	193.11 ± 3.26†††	196.67 ± 3.2**†††			
5 minutes[a]	117.5 ± 10.28†††	117.5 ± 11.98†††	106.56 ± 15.3†††	111.56 ± 14.44†††			
10 minutes[a]	103.8 ± 8.61†††	101.2 ± 7.79†††	95.11 ± 13.13††	95.56 ± 12.69†††			
**Anaerobic capacity**							
**Blood lactate (mmol/L)**					0.002 (0.219)	<0.001 (0.707)	0.922 (0.014)
Resting	2.12 ± 0.98	3.44 ± 2.45	2.24 ± 0.86	2.83 ± 1.1			
0 (Immediately)[b]	12.47 ± 5.33†††	11.68 ± 3.74†††	10.03 ± 1.93†††	7.31 ± 2.67#†			
5 minutes[b]	11.95 ± 3.54	9.87 ± 3.04*	11.99 ± 3.14	11.38 ± 2.02			
7 minutes[b]	9.96 ± 2.94	9.39 ± 2.77	10.16 ± 1.55	11.21 ± 1.7			
9 minutes[b]	7.84 ± 2.11	7.98 ± 1.55	8.73 ± 2.01	9.47 ± 1.34#			

[a]represents after exhaustion;

[b]represents after exercise;

Significant difference within Group (*p < 0.05, **p < 0.01);

Significant difference between group (#p < 0.05);

Significant difference compared to the previous time point (†p < 0.05, ††p < 0.01, †††p < 0.001)

Table S6. Differential metabolites screened according to predetermined criteria

PEP group					PRO group				
**Metabolite**	**FC**	**FDR**	**VIP**	**Trend**	**Metabolite**	**FC**	**FDR**	**VIP**	**Trend**
**23-Norcholic Acid**	1.580	0.007	1.559	↑	**7-Ketolithocholic Acid Methyl Ester**	2.008	0.000	1.466	↓
**23-Norcholic Acid Diacetate**	1.648	0.006	1.640	↑	**Acetoacetic acid**	1.537	0.002	1.057	↑
**7-Ketolithocholic Acid Methyl Ester**	0.368	0.012	1.552	↓	**D-Xylose**	2.371	0.002	1.785	↑
**4-ANDROSTEN-3-ONE-17beta-CARBOXYLIC ACID**	0.496	0.028	1.297	↓	**2'-Deoxyadenosine**	1.542	0.002	1.178	↓
**N-(4-Aminobenzoyl)-L-glutamic Acid**	1.648	0.032	1.311	↑	**Oxalacetic acid**	0.439	0.002	2.160	↑
**3-Hydroxybutyric acid**	0.535	0.018	1.391	↓	**3-Methyladipic acid**	1.570	0.002	1.281	↓
**D-Xylose**	1.555	0.027	1.382	↑	**Cholic Acid**	0.634	0.003	1.294	↓
**Oxalacetic acid**	1.756	0.010	1.732	↑	**Dodecanedioic acid**	2.605	0.004	1.405	↓
**7-Ketolithocholic Acid**	1.546	0.013	1.585	↑	**alpha-Hydroxyisobutyric acid**	0.643	0.004	1.588	↑
**5Z-Dodecenoic acid**	0.503	0.018	1.255	↓	**Dehydrolithocholic acid**	2.060	0.004	1.463	↑
**alpha-Hydroxyisobutyric acid**	1.573	0.010	1.374	↑	**Alpha-Linolenic acid**	1.679	0.010	1.406	↓
**Hydroxyisocaproic acid**	1.509	0.014	1.599	↑	**Pyridoxal**	1.724	0.012	1.858	↑
**3-Hydroxyisovaleric acid**	0.742	0.028	1.458	↓	**Aspartame**	0.674	0.016	1.450	↑
**Alpha-Linolenic acid**	0.291	0.006	1.855	↓	**Myristoleic acid**	0.467	0.017	1.464	↓
**Aspartame**	1.746	0.019	1.475	↑	**11Z-Eicosenoic acid**	1.629	0.021	1.129	↓
**Myristoleic acid**	0.349	0.007	1.578	↓	**8,11,14-Eicosatrienoic acid**	0.470	0.021	1.831	↓
**11Z-Eicosenoic acid**	0.191	0.005	1.860	↓	**Shikimic acid**	0.384	0.025	1.738	↑
**Lauroylcarnitine**	0.492	0.023	1.326	↓	**Quinic acid**	0.704	0.025	1.671	↓
**8,11,14-Eicosatrienoic acid**	0.531	0.006	1.716	↓	**Palmitelaidic acid**	0.354	0.029	1.594	↓

**Shikimic acid**	2.306	0.027	1.545	↑	**Glycylvaline**	0.481	0.033	1.813	↑
**Quinic acid**	0.348	0.005	1.856	↓	**(S)C(S)S-S-Methylcysteine sulfoxide**	0.476	0.033	1.263	↓
**11C,14C-EICOSADIENOIC ACID**	0.635	0.032	1.302	↓	**trans-Piceid**	1.564	0.036	1.731	↑
**Adipoylcarnitine**	0.656	0.028	1.373	↓	**2-Methylvaleric acid**	0.715	0.041	1.421	↑
**Palmitelaidic acid**	0.399	0.010	1.543	↓	**11-Dodecenoic acid**	0.683	0.042	1.301	↓
**10Z-Nonadecenoic acid**	0.633	0.028	1.429	↓	**10Z-Heptadecenoic acid**	0.550	0.048	1.347	↓
**Glycylvaline**	2.003	0.023	1.558	↑	**CIS-11,14,17-EICOSATRIENOIC ACID**	0.472	0.049	1.848	↓
**Leucyl-Glycine**	1.742	0.035	1.441	↑	**Myristelaidic acid**	0.591	0.049	1.455	↓
**2-Methylvaleric acid**	1.699	0.016	1.615	↑					
**11-Dodecenoic acid**	0.475	0.023	1.315	↓					
**Undecylenic acid**	0.528	0.023	1.289	↓					
**10Z-Heptadecenoic acid**	0.410	0.008	1.581	↓					
**CIS-11,14,17-EICOSATRIENOIC ACID**	0.526	0.006	1.749	↓					
**O-Adipoylcarnitine**	0.714	0.048	1.160	↓					
**Myristelaidic acid**	0.346	0.007	1.575	↓					

FC = fold change; FDR = false discovery rate; VIP = Variable Importance in Projection; ↑ represents upregulate; ↓ represents downregulate
